# Liver Xenotransplantation: A Path to Clinical Reality

**DOI:** 10.3389/ti.2024.14040

**Published:** 2025-01-03

**Authors:** Serkan Sucu, Yucel Yankol, Luis A. Fernandez, Burcin Ekser

**Affiliations:** ^1^ Division of Transplant Surgery, Department of Surgery, Stritch School of Medicine, Loyola University Chicago, Maywood, IL, United States; ^2^ Department of Surgery, Koc University School of Medicine, Istanbul, Türkiye

**Keywords:** coagulation dysregulation, liver, pig, platelets, xenotransplantation

## Abstract

Liver xenotransplantation has emerged as a potential solution to the shortage of deceased human donor organs and is now becoming a reality due to recent developments in genetic engineering and immunosuppressive therapy. Early efforts using non-human primates and genetically modified pigs faced significant challenges such as thrombocytopenia and graft rejection. Understanding the mechanism behind those challenges and using novel genetically engineered pigs enabled researchers to overcome some of the hurdles, but more research is needed. However, new advances might allow pig liver xenotransplantation to potentially serve as a bridge to liver allotransplantation or allow native liver regeneration in the near future.

## Introduction

Liver diseases are a significant cause of morbidity and mortality. Although advances in antiviral therapy have decreased morbidity and mortality from viral hepatitis, the number of patients with non-alcoholic fatty liver disease is increasing significantly due to the rising obesity epidemic. According to National Health and Nutrition Examination Surveys, its prevalence has risen from 20% between 1988 and 1994 to 32% between 2009 and 2012 [1]. Moreover, the prevalence of alcoholic liver disease is rising due to increased alcohol consumption in the world [2]. As a result, the incidence of end-stage liver disease and related cirrhosis has substantially increased which has accentuated the donor liver shortage.

Liver transplantation provides a potential cure for patients with end-stage liver disease. However, due to the limited availability of organ donors, a significant number of patients die on the waiting list from complications related to cirrhosis [3]. In addition to the scarcity of organ donors with a transplantable liver, the allocation and transportation of liver allografts pose another significant burden. According to the United Network for Organ Sharing (UNOS) of the United States, the median waiting time for the most urgent liver transplant listing, which is considered a national emergency (Liver Status 1A) in cases of acute or fulminant liver failure, is 6 days. In the case of acute liver failure, only 55% of patients survive without liver transplantation [4]. Between 2011 and 2014, the median time for a non-urgent listing for liver transplantation for patients aged between 18 and 34 years was 1,048 days, for those 35–49 years was 1,080 days, and for those 50–64 years was 1,136 days [5]. After a recent change in liver allocation, the median waiting times are yet to be announced by the UNOS.

Liver transplantation is now offered as a new treatment option for patients with unresectable liver metastasis from colorectal cancer, which causes an increase in demand while supply stays almost the same [6]. In fact, there have been >50,000 patients who either died or were removed from the waiting list for being ‘too sick to transplant’ within the last 20 years [5].

Xenotransplantation, which is the transplantation of organs between different species, offers a potential solution to the scarcity of organs, providing a potentially unlimited supply of organs [7]. However, xenotransplantation brings challenges because of immunological barriers, interspecies incompatibility, coagulation dysregulation, and ethical problems. Advances in genetic engineering and improved immunosuppressive treatments have allowed us to overcome many challenges [8]. In this review, we summarize recent progress in clinical xenotransplantation, and in overcoming the immunological barriers and coagulation dysregulation (with a special focus on thrombocytopenia and coagulopathy), as well as a brief discussion of the ethical considerations. We also provide a summary of previously performed clinical liver xenotransplantation as a basis for creating a path to clinical liver xenotransplantation in the near future.

## Recent Progress in Clinical Organ Xenotransplantation

Recently, there have been multiple significant developments in clinical xenotransplantation. The first (in 2022) was a pig-to-human cardiac xenotransplant using a 10-gene genetically-engineered pig heart and a novel co-stimulation blockade agent, anti-CD40 monoclonal antibody (mAb), carried out at the University of Maryland at Baltimore [9]. The recipient of the pig heart lived for 61 days. The first clinical pig kidney transplant was carried out at the Massachusetts General Hospital in 2024. A kidney from a similar gene-edited pig was transplanted into a 62-year-old male patient with end-stage renal disease who lived for 52 days before dying of a cardiac complication ([10]; Kawai T et al, manuscript submitted).

### Initial Experience in Clinical Liver Xenotransplantation

Developments in liver xenotransplantation have been slower even though, given its emergency and severity nature, acute liver failure requires urgent attention. One key reason is that liver xenotransplantation is more challenging than kidney and heart xenotransplantation due to major problems with thrombocytopenia and coagulopathy in addition to the immunological barriers to xenotransplantation [11].

Initial efforts to transplant livers from non-human primates (NHPs) into human recipients began in the 1960s (Table 1). Allotransplant pioneer, Thomas Starzl, and his colleagues performed three chimpanzee liver transplants in humans between 1966 and 1974, with the grafts functioning from only 1–14 days [21]. However, in 1993, he achieved a significantly longer survival when a 35-year-old man with chronic hepatitis B and HIV infection received a liver transplant from a baboon (Table 1) [22]. The surgical procedure followed conventional techniques, with immunosuppression using a regimen of tacrolimus, prednisone, prostaglandin, and a non-myelotoxic dose of cyclophosphamide. Despite the challenges of transplantation between species, the patient showed little evidence of hepatic rejection, with biochemical monitoring and histopathological examination indicating effective liver function for 70 days post-transplantation. The transplanted baboon liver produced clotting factors and other proteins without adverse effects. Unfortunately, the patient died of a cerebral hemorrhage due to an invasive aspergillus infection, but there was underlying widespread biliary sludge formation in the biliary tree [22].

**TABLE 1:** World experience with clinical liver xenotransplantation (1966–2024).

Surgeon/Year	Recipient age/sex (M = male, F = female)	Donor animal	Underlying pathology	Surgical technique	Preventive measures and immunosuppressive therapy	Outcome
Starzl/1966 [12]	28 months/M	Chimpanzee	Intrahepatic biliary atresia	OLT	Azathioprine, prednisone, antithymocyte globulin	Evidence of function was seen in hepatic xenograft, the patient was lost at POD 9 due to severe infections from excessive immunosuppression
Starzl/1969 [13]	7 months/M	Chimpanzee	Extrahepatic biliary atresia	OLT	Liver transplantation with both donor kidneys, receiving systematic arterial supply and draining to portal system	Death at 26th hour from brain edema, child never woke up from surgery. There was evidence of liver xenograft function postoperatively and no evidence of hepatocellular damage on autopsy
Bertoye/1969 [14]	22 years/F	Baboon	NA	HLT	NA	Survived for more than 4 months
Bertoye/1969 [14]	7 months/M	Baboon	NA	HLT	NA	Died at 39th hour after surgery
Marion/1970 [15]	NA/NA	Baboon	NA	HLT	NA	Survived less than 24 h
Leger/1970 [16]	23 years/F	Baboon	NA	HLT	NA	Survived for 72 h
Pouyet/1971 [17]	28 years/F	Baboon	NA	HLT	NA	Survived for less than 2 days
Pouyet/1971 [16]	34 years/F	Baboon	NA	HLT	NA	Survived for less than 2 days
Motin/1971 [16]	NA/NA	Baboon	NA	HLT	NA	Survived for 3 days
Starzl/1974 [18]	23 months/NA	Chimpanzee	Persistent acute rejection following liver allotransplantation (for biliary atresia)	OLT	NA	Survived 14 days. No evidence of hyperacute rejection. Centrilobular cholestasis
Starzl/1992 [18]	35 years/NA	Baboon	Decompensated cirrhosis from hepatitis B infection	OLT	Tacrolimus, cyclophosphamide	Death from subarachnoid hemorrhage caused by invasive aspergillus infection at day 70
Starzl/1993 [18]	62 years/M	Baboon	Decompensated cirrhosis from hepatitis B infection	OLT	Tacrolimus, cyclophosphamide	Anastomotic leak and bile peritonitis at day 26
Makowka/1993 [19]	26 years/F	Wild-type pig	Fulminant hepatic failure from autoimmune hepatitis	HLT	Plasmapheresis	Rapid rise in xenoantibody levels and liver failure after 3 h
Beicheng/2024 [20]	71 years/M	10-gene pig	Large liver cancer	HLT	NA	After 2 weeks, patient had good liver function. No further information

The table was modified from Taniguchi and Cooper [17].

HLT, heterotopic liver transplantation; NA, not available; OLT, orthotopic liver transplantation; POD, post-operative day.

One of the most critical steps in xenotransplantation research was realizing that NHPs are not ideal sources of organs for clinical xenotransplantation [23]. Pigs are better candidates than NHPs for several reasons, including their similarity in size, anatomy, and physiology, though, unless genetically-engineered, they are at a distinct disadvantage immunologically. Most recently in 2024, a genetically engineered (10-gene-edited) pig liver was transplanted into a 71-year-old living male patient who suffered from a large liver tumor that could be removed but would leave him with an inadequate remnant of native liver that would not sustain life [20]. Therefore, a 514-gram whole pig liver was transplanted in an auxiliary fashion to augment the function of the remainder of the native liver tissue while it regenerated and grew. Although the surgeons reported that the patient was doing well with good liver function for 2 weeks after the clinical pig liver xenotransplantation, no further formal scientific data have yet been reported [20].

## Immunological Barriers

After identifying a suitable species as a source of organs for clinical xenotransplantation (the pig), the next major challenge was overcoming the immunological barriers. The innate and adaptive immunological barriers to xenotransplantation have been discussed previously by others [24]. Briefly, the innate immunological barriers include hyperacute rejection, associated with the presence of natural anti-pig (mainly directed to Gal antigens – see below) antibodies, complement activation, neutrophil infiltration, NK cell-mediated cytotoxicity, and macrophage activation [24]. The adaptive response includes the T cell response, including direct recognition of MHC class I on xenograft cells by CD8^+^ and CD4^+^ T cell activation, leading to proinflammatory cytokine release, and B cell activation [25]. Disruption of endothelial cells and dysregulation of the coagulation cascade caused by immunological activation are among the major challenges of pig liver xenotransplantation [24].

The initial focus of xenotransplant research was genetically modifying the pig to overcome the complement response, especially with the expression of humanized complement-regulatory proteins (CD55 or decay-accelerating factor) [11, 26, 27]. However, the identification of galactose-α-1,3-galactose (Gal), the major antigen present in pigs (but absent in humans), was among the most significant steps in xenotransplantation research [28–30]. Galili et al. and Good et al. independently demonstrated that humans have naturally existing anti-Gal antibodies that bind to Gal antigens on pig vascular endothelial cells, which was crucial for understanding the mechanism of hyperacute rejection [28–30]. When this was known, it was suggested that expression of Gal in pigs should be deleted by gene editing [31], although this was not possible at that time. It was not until a decade later that this significant problem was overcome through genetic engineering with the generation of α1,3-galactosyltransferase gene-knockout (GTKO) pigs [32–34].

There was a marked increase in the survival of NHPs that received a heart or kidney from a GTKO pig [35, 36]. GTKO pigs prevented hyperacute rejection and extended pig heterotopic (non-life-supporting) heart survival in baboons for up to 6 months [35]. With induction therapy, including at that time thymectomy, splenectomy, and T cell depletion, and maintenance therapy with an anti-CD154 monoclonal antibody (mAb), and mycophenolate mofetil with or without low-dose steroids, life-supporting kidney xenografts survived for up to 83 days with normal creatinine levels [36]. Similar success was anticipated from liver xenotransplantation, but this was not the case.

### Pig-to-NHP Liver Xenotransplantation

Initial studies that used genetic modification technology in liver xenotransplantation were reported by Ramirez et al. [27]. The livers came from pigs expressing human CD55 to protect from complement activation. Two recipient NHPs survived for 4 and 8 days, but thrombocytopenia and coagulopathy were significant challenges [27]. Liver xenografts from GTKO pigs or GTKO pigs transgenic for human CD46 were implanted in baboons and survived for up to 7 days [37]. Even though the baboons died from bleeding associated with thrombocytopenia, liver function, including coagulation and synthetic functions, were within an acceptable range [37]. Using GTKO pigs and demonstrating adequate hepatic function helped researchers focus on the next problem, which was thrombocytopenia.

## Thrombocytopenia

**FIGURE 1:**
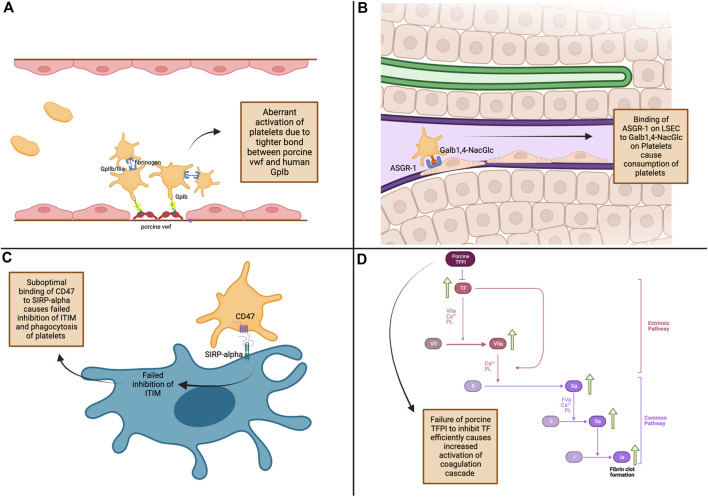
Mechanisms of thrombocytopenia and coagulation dysregulation in pig-to-non-human primate liver xenotransplantation. **(A)** - Platelet Activation: Tight binding of porcine von Willebrand factor (vWF) to human GpIb receptors leads to increased platelet activation [11]. **(B)** - Platelet Consumption: ASGR1 on porcine liver sinusoidal endothelial cells binds to Galβ1,4-NacGlc on human platelets, causing platelet consumption [39]. **(C)** - Failed Phagocytosis Inhibition: Inadequate binding of porcine SIRP-α to human CD47 leads to phagocytosis of platelets [19]. **(D)** - Coagulation Cascade Activation: Porcine TFPI inefficiently inhibits tissue factor, leading to increased coagulation and fibrin clot formation [41]. ASGR1, asialoglycoprotein receptor-1; CD47, Cluster of Differentiation 47; Galb1,4-NacGlc, galactose β1-4 N-acetyl glucose; GpIIb/IIIa, glycoprotein IIb/IIIa; GpIb, glycoprotein Ib; ITIM, iImmunoreceptor tyrosine inhibitory motifs; LSEC, liver sinusoidal endothelial cells; SIRP-α, signal regulatory protein α; TF, tissue factor; TFPI, tissue factor pathway inhibitor; vWF, von Willebrand factor.

Various mechanisms have been proposed for the thrombocytopenia that occurs in liver xenotransplantation, such as 1) tighter binding of porcine von Willebrand factor (vWF) to human platelet glycoprotein Ib (GpIb) causing aberrant platelet activation, 2) increased platelet sequestration and phagocytosis by porcine Kupffer cells as well as non-function of the “do not eat me” signal due to incompatibility between porcine CD47 and human SIRPalpha, 3) platelet consumption by porcine liver sinusoidal endothelial cells via asialoglycoprotein receptor-1, and 4) failure of porcine tissue factor pathway inhibitor to inhibit human tissue factor, causing aberrant activation of the coagulation cascade [11], [38–41] (Figure 1).

Burlak et al. demonstrated that thrombocytopenia was a significant problem when they perfused pig livers with human platelets, showing that 93% of the platelets were removed from the circulation within 15 min [42]. When baboons received orthotopic liver xenografts from GTKO pigs transgenic for CD46, severe thrombocytopenia developed within 1 h (although the recipient baboons survived for several days) [43]. To overcome lethal thrombocytopenia in liver xenotransplantation, Kim et al. used aminocaproic acid and achieved 9 days of survival of GTKO pig livers in baboons [44]. Even though there was no evidence of rejection, and aminocaproic acid treatment was successful at keeping the number of platelets at >40,000/mL, the recipients still died or were euthanized for uncontrolled bleeding [44]. As a result, efforts to overcome thrombocytopenia and coagulopathy continued in liver xenotransplantation research.

A recent explanation for thrombocytopenia is species incompatibility in platelet glycosylation. Burlak et al. showed that platelet alpha granules contain IgM and IgG antibodies targeting xenoantigens, including Gal, Neu5Gc, and Sda. This leads to thrombocytopenia due to platelet phagocytosis by porcine liver sinusoidal endothelial cells, activation of the immune response against platelets, and tethering of platelets in the xenograft [45].

Kupffer cells also play a role in thrombocytopenia seen in xenotransplantation. Mechanisms such as the Mac-1–β-glucan pathway, as well as CD40–CD40L, and CD18–CD40L interactions, are shown to be contributors to platelet phagocytosis. The Mac-1 (CD11b/CD18)-β-glucan interaction enables pig Kupffer cells to recognize and phagocytose desialylated platelets, with exposed N-acetyl D-glucosamine (β-GlcNac) acting as a binding ligand. This interaction triggers phagocytosis of platelets, leading to their rapid depletion [42]. In addition, CD18, which is a part of integrin heterodimer expressed on pig Kupffer cells, recognizes CD40L on human platelets, which were activated due to the inflammatory and immunological environment created during liver xenotransplantation [11]. CD40L is also upregulated on the surface of platelets following activation. This interaction causes phagocytosis of platelets by pig Kupffer cells. Finally, pig Kupffer cells express CD40, and their interaction with CD40L on human platelets triggers immune activation, leading to the release of proinflammatory cytokines, platelet adhesion, and phagocytosis, all of which contribute to thrombocytopenia [11].

## Coagulopathy

Transfusion of human coagulation factors and immunosuppressive regimens improved the survival of the liver xenograft, but underlying pathophysiological mechanisms persisted [11]. The underlying mechanisms postulated for coagulation dysfunction include pre-existing anti-nonGal antibodies causing tissue factor expression on pig endothelial cells, cross-species molecular incompatibilities in the protein C pathway, incompatibility between porcine vWF and human platelet GPIb, incompatibility between porcine tissue factor pathway inhibitor and human factor Xa, procoagulant tissue factor expression by xenograft endothelial cells, and direct prothrombinase activity of fibrinogen-like protein 2 in xenograft endothelium [46, 47].

To counteract coagulation abnormalities and thrombocytopenia, Navarro-Alvarez et al. administered exogenous human prothrombin concentrate complex, including factors II, VII, IX, X, Protein C and S or Factor VIIa, to baboons that received liver xenografts from GTKO pigs [48]. Even though the recipients were lost by postoperative day 7, decreased transfusions were required in baboons that received exogenous human coagulation factor concentrate. Moreover, those that received Factor VII showed an increase in circulating platelet counts. There was no thrombotic microangiopathy in recipient baboons receiving human coagulation factors, in contrast to previous studies. With 1) the addition of co-stimulation blockade with belatacept or anti-CD40mAb, 2) selection of CMV-negative pig donors, and 3) exogenous use of human coagulation factors, Shah et al. reported 25 days and 29 days of survival following GTKO pig liver xenotransplantation in baboons, with recovery of low platelet counts after postoperative days 7 or 8 [49, 50]. The remarkable survival of two baboons for almost 4 weeks made it possible to anticipate that liver xenografts might be used for bridging to allotransplantation.

Among these mechanisms, the role of thrombomodulin in regulating the coagulation cascade has also been highlighted as a significant contributor to coagulopathy in xenotransplantation. Thrombomodulin, a key component of the coagulation cascade, acts as a cofactor for thrombin and converts thrombin from a pro-coagulant enzyme to an anticoagulant by activating protein C, which subsequently inhibits clot formation. One of the proposed mechanisms in coagulopathy seen in xenotransplantation is the fact that human thrombomodulin may not effectively interact with porcine thrombin due to structural differences, leading to impaired anticoagulant activity and increased risk of coagulopathy. In particular, the interspecies thrombin-thrombomodulin complex has been shown to inadequately activate human protein C, contributing to impaired anticoagulation and leading to coagulopathy [11].

## Ethical Considerations

While scientific and clinical progress in xenotransplantation provides a new solution to the organ shortage, it also brings to the forefront a range of ethical considerations that must be carefully navigated. Pigs that are raised for organ transplantation are not kept in traditional husbandry conditions; instead, they are kept in clean biosecure (designated pathogen-free) conditions that minimize the risk of pathogen proliferation. This may possibly cause some psychological insecurity that may be detrimental to animal welfare [51].

The use of animal organs, particularly from pigs, can conflict with religious and cultural beliefs and practices, raising issues of respect, dignity, and the need for sensitivity in the medical community. However, when it is a question of life or death, Judaism allows its believers to violate this rule to save human life [52]. In a theological symposium organized by the International Xenotransplantation Association in 2017, the issue of religious beliefs in relation to xenotransplantation was broadly discussed. In the presence of Christian, Jewish, and Muslim representatives, theological perceptions and perspectives about xenotransplantation, the genetic alterations in the organ-source pig, and their acceptance by individuals were explored towards potential clinical trials [53]. There was no absolute opposition to clinical xenotransplantation.

## Comments and Conclusion

Even though major challenges remain to be overcome if liver xenotransplantation is to become successful destination therapy, pig-to-NHP liver survival has been extended to 29 days. Pig liver xenografts may therefore play a life-saving role initially as a bridge to allotransplantation for patients with high risk of mortality [50, 54].

An increase in the survival of experimental liver xenografts has directed more attention to the indications for clinical liver xenotransplantation. There are scarce examples of clinical liver xenotransplantation from diverse sources of animals (Table 1). Today, it is unlikely that the use liver xenografts from NHPs would be approved [12–17].

Of the two cases of pig-to-human liver xenotransplantation that have been carried out, one was performed using a wild-type pig liver and, despite the removal of xeno-antibodies by plasmapheresis and wild-type pig kidney perfusion, the level of xeno-antibodies increased rapidly, inducing rejection which limited graft survival to a few hours (Table 1). To date, the outcome of the second pig-to-human liver xenotransplant using a 10-gene pig liver has not yet been reported scientifically.

Even though it might be premature, more experience gleaned from clinical experiments on “compassionate” grounds might soon justify initial formal clinical trials of bridging to allotransplantation in selected patients with acute liver failure or “liver xenograft bridge-to-regeneration of native liver” in selected liver cancer patients (primary or metastases) where the native liver remnant is insufficient to sustain life.

## Abbreviations

CMV, cytomegalovirus; GTKO, α1,3-galactosyltransferase gen-knockout; hDAF, human decay-accelerating factor; mAb, monoclonal antibody; NHP, non-human primate.
